# IL-17B Can Impact on Endothelial Cellular Traits Linked to Tumour Angiogenesis

**DOI:** 10.1155/2010/817375

**Published:** 2010-05-06

**Authors:** Andrew J. Sanders, Xiaoxia Guo, Malcolm D. Mason, Wen G. Jiang

**Affiliations:** ^1^Metastasis and Angiogenesis Research Group, Cardiff University School of Medicine, Heath Park, Cardiff CF14 4XN, UK; ^2^Department of Clinical Oncology, Cardiff University School of Medicine, Heath Park, Cardiff CF14 4XN, UK

## Abstract

IL-17B is a member of the IL-17 cytokine family which have been implicated in inflammatory response and autoimmune diseases such as rheumatoid arthritis. The founding member of this family, IL-17 (or IL-17A), has also been implicated in promoting tumour angiogenesis through the induction of other proangiogenic factors. Here we examine the potential of recombinant human IL-17B to contribute to the angiogenic process. In vitro rhIL-17B was able to inhibit HECV endothelial cell-matrix adhesion and cellular migration and also, at higher concentrations, could substantially reduce tubule formation compared to untreated HECV cells in a Matrigel tubule formation assay. This data suggests that IL-17B may act in an antiangiogenic manner.

## 1. Introduction

IL-17, initially termed CTLA-8, was identified in 1993 through subtractive hybridisation of a rodent cDNA library and was found to share homology to the ORF13 gene of the Herpesvirus saimiri. Subsequent studies identified both the receptor for IL-17/CTLA-8 cytokine and the human version of IL-17. IL-17 has been shown to induce IL-6 and IL-8 production together with enhanced expression of ICAM-1 and NF-*κ*B activity [1–3]. Since their initial discovery, research into the IL-17 cytokine family has identified six members, IL-17 (IL-17A) -IL-17F, which signal through a family of IL-17 receptors, IL-17RA–IL-17RE (reviewed in full in [4, 5]). 

IL-17 can cause the induction of proinflammatory factors, contributing to the immune response. However this cytokine has also been shown to play a role in autoimmune disorders such as rheumatoid arthritis [6–9]. Various studies have also investigated the role of IL-17 in cancer progression, discovering that IL-17 possesses both anti- and pro tumour roles. IL-17-transfected haematopoietic tumours grafted onto syngeneic immunocompetent mice grew at a significantly reduced rate to mock transfected control tumours and this reduction was associated with the enhanced generation of CTLs [10]. Another study suggests that endogenous IL-17 may be involved in tumour immunity and show that MC38 colon cancer cells inoculated subcutaneously developed significantly faster in IL-17-deficient mice than wild-type mice and developed significantly more metastatic foci than wild-type mice following intravenous injection of MC38 [11]. The protumorigenic effects of IL-17 seem largely due to its ability to contribute to the inflammatory response and enhance angiogenesis. IL-17-transfected tumour cells have been shown to enhance in vivo growth and show significantly enhanced tumour vascularity compared to control cells, whilst in vitro IL-17 treatment stimulated vascular endothelial cell migration and cord formation and caused upregulation of a number of proangiogenic factors in fibroblasts and tumour cells [12]. Subsequent work has shown that IL-17 could enhance HGF-, VEGF-, and bFGF- induced vascular endothelial cell growth and thus may help to mediate angiogenesis promoted through these growth factors [13]. Elevated levels of serum IL-17 have been detected in multiple myeloma patients, with levels being significantly higher in stage II and III patients than in stage I patients and in these patients serum IL-17 levels were found to correlate positively with levels of VEGF, TNF*α*, and micro vessel density [14].

Currently there are few studies focusing on the IL-17B member of the IL-17 cytokine family in cancer progression and angiogenesis. IL-17B was initially cloned and characterised in 2000 [15]. IL-17B shares approximately 27% amino acid identity with IL-17, has a wider expression pattern with transcripts being demonstrated in human adult pancreas, small intestine, stomach, and testis, and can stimulate the release of tumour necrosis factor alpha (TNF*α*) and IL-1*β* in the THP-1 monocytic cell line [15]. IL-17B has also been suggested to play an important role in inflammatory arthritis [16]. In the current study we use rhIL-17B to assess the role of IL-17B in the human HECV endothelial cell line and its potential to impact on traits, such as cellular migration and tubule formation, associated with the angiogenic process.

## 2. Methodology

### 2.1. Reagents, Cell Lines, and Culture Conditions

The human HECV endothelial cell line was purchased from the European Collection of Animal Cell Cultures (ECACC, Salisbury, UK). Cells were routinely subcultured in Modified Eagle Medium (DMEM) (PAA Laboratories Ltd., Somerset, UK) supplemented with 10% Fetal Bovine Serum (PAA Laboratories Ltd, Somerset, UK), penicillin, and streptomycin. The cells were maintained in an incubator at 37°C and 5% CO_2_ humidity. Recombinant human IL-17B (rhIL-17B) was purchased from R&D systems (Abingdon, UK).

### 2.2. Cell Function Assays

#### 2.2.1. In Vitro Cell Growth Assay

The impact of rhIL-17B on HECV cell growth was assessed using an in vitro cell growth assay. Cells were seeded into a 96-well plate at a seeding density of 3,000 cells/well and treated with a range of rhIL-17B concentrations (0 ng/mL, 10 ng/mL, 50 ng/mL, 100 ng/mL, and 250 ng/mL). Triplicate plates were set up and incubated for periods of overnight, 3 days or 5 days. Following incubation, the medium was removed; cells were fixed in 4% (v/v) formalin for 5 minutes, stained in 0.5% (w/v) crystal violet for 5 minutes, and rinsed. The crystal violet stain taken up by the cells was then extracted in a 10% (v/v) acetic acid solution and cell growth was determined spectrophotometrically using a Bio-Tek ELx800 multiplate reader (Bio-Tek Instruments Inc., Vermont, USA).

#### 2.2.2. In Vitro Matrigel Adhesion Assay

The impact of rhIL-17B on HECV cell-matrix adhesion was assessed using an in vitro Matrigel adhesion assay previously described [17]. In brief, wells of a 96-well plate were precoated with 5 *μ*g of Matrigel (BD Biosciences, Oxford, UK) before seeding 30,000 HECV cells in medium containing a range of rhIL-17B concentrations (0 ng/mL control, 10 ng/mL, 50 ng/mL, and 100 ng/mL). Cells were incubated for 45 minutes before being subjected to intense washing to remove nonadherent cells, fixed in 4% (v/v) formalin, and stained with 0.5% (w/v) crystal violet. Adherent cells were subsequently observed under a microscope and the number of adherent cells per field were calculated in a number of random fields. 

#### 2.2.3. In Vitro Migration/Wounding Assay

A migration/wounding assay was used to assess the impact of rhIL-17B on HECV cellular migration. This protocol was modified from a previously described method [18]. Briefly, cells were cultured in a 24-well plate until they reached a near confluent monolayer. This monolayer was subsequently scratched with a 21G needle and the medium was replaced with fresh medium containing a range of concentrations of rhIL-17B (0 ng/mL control, 10 ng/mL, 50 ng/mL, and 100 ng/mL). Cellular migration from the two wound fronts was tracked and recorded over a 90-minute period using a time-lapse video recorder (Panasonic, Japan). Migration rates were those calculated at 15 minute-time intervals within the 90-minute period using Optimas 6 motion analysis software.

#### 2.2.4. In Vitro Matrigel Tubule Formation Assay

Matrigel endothelial cell tube formation assays were set up to assess any impact on angiogenic effect following treatment with rhIL-17B at a range of concentrations (0 ng/mL, 10 ng/mL, 50 ng/mL, 100 ng/mL, and 250 ng/mL). The protocol used was modified from that previously reported [19]. Briefly, 250 *μ*g of Matrigel was seeded into a 96-well plate in serum-free medium and left to gel in an incubator for a minimum of 40 minutes. Once the Matrigel had set, 30,000 HECV cells were seeded onto the Matrigel layer and incubated for 4-5 hours to allow tubule formation to occur. Following incubation, any tubules that had formed were visualized under low magnification and images captured. Total tubule perimeter/field in these images were later quantified using ImageJ software. 

### 2.3. Statistical Analysis

All experimental procedures were repeated a minimum of three independent times. Data was analysed using Minitab 14 statistical package using a two-sample two-tailed *t*-test to compare treated samples to that of untreated controls. Values of *P* < .05 were taken as being statistically significant.

## 3. Results and Discussion

### 3.1. rhIL-17B Had Little Impact on HECV Cell Growth over a Range of Concentrations

**Figure 1:**
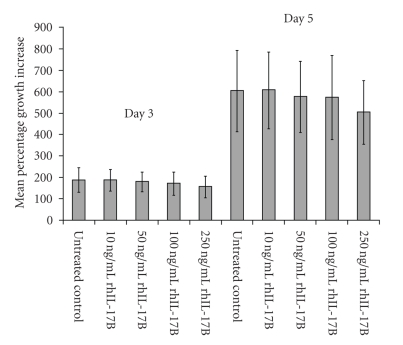
HECV growth was not affected by rhIL-17B treatment. No significant difference in HECV growth rate was observed following 3- or 5-day incubation periods between the untreated (0 ng/mL) control and any other rhIL-17B treatment concentration (10 ng/mL, 50 ng/mL, 100 ng/mL, and 250 ng/mL).

rhIL-17B was added to the cells at concentrations of 10 ng/mL, 50 ng/mL, 100 ng/mL, and 250 ng/mL and the growth rates were compared to those an untreated control in order to determine the toxicity of rhIL-17B. Treatment of HECV cells with rhIL-17B seemed to have little effect on the growth rate of this cell line over the incubation period and concentrations tested (Figure 1). Following 3-day incubation at the various treatments, there is very little variation between the growth rates of the treated cells and the untreated control cells. Following a longer 5-day treatment of HECV cells with rhIL-17B, a noticeable drop in growth can be seen in the highest tested concentration of 250 ng/mL; however all other treatment concentrations show little variability in growth rates compared to untreated control HECV cells. No significant difference was observed at any concentration at any specific incubation period when compared to the untreated control of the same incubation period. This suggests that IL-17B, at the tested concentrations, does not play a significant role in the regulation of cell growth in the HECV human endothelial cell line.

### 3.2. rhIL-17B Negatively Impacted on HECV Cell-Matrix Adhesion and Migration

**Figure 2:**
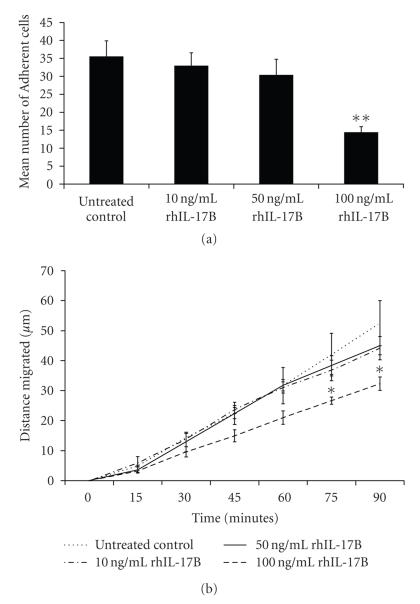
(a) Effect of rhIL-17B on HECV cell-matrix adhesion. A significant reduction in cell-matrix adhesion was observed following treatment with 100 ng/mL rhIL-17B. (b) Effect of rhIL-17B on HECV cell migration. Similarly, 100 ng/mL rhIL-17B significantly inhibited HECV cell migration and a significant difference compared to the untreated control in migration was observed following 75- and 90-minute periods. *P < .05; **P < .01.

Treatment of HECV endothelial cells with rhIL-17B could influence both cell-matrix adhesion (Figure 2(a)) and cellular migration (Figure 2(b)). Lower concentrations of rhIL-17B (10 ng/mL and 50 ng/mL) had minimal effect on these two traits; however at the higher concentration of 100 ng/mL a significant reduction in both cell-matrix adhesion and cellular migration in comparison to untreated control HECV cells was noticed. Treatment of HECV cells with 100 ng/mL rhIL-17B reduced the capacity of the cells to adhere to an artificial Matrigel basement membrane (*P* = .004). Similarly, at this concentration, rhIL-17B significantly reduced cell migration over a 90-minute period where the migration rates of cells treated with 100 ng/ml rhIL-17B were significantly lower than those of the untreated control cells after 75 minutes (*P* = .048) and at the experimental endpoint of 90 minutes (*P* = .027). Thus, it appears that IL-17B, at higher concentrations, may play a role in regulating HECV cell-matrix adhesion and cell migration. A previous study has investigated the role of IL-17 in endothelial cell migration in vitro, using a modified Boyden chamber assay system, and demonstrated a promotional effect of IL-17 on endothelial cell migration [12]. Our study examined the impact of rhIL-17B on endothelial cell migration using a wounding cell migration system, and whilst differing methodology makes it difficult to draw direct comparisons between IL-17 and IL-17B, our results indicate a potential role for IL-17B in the migration of HECV endothelial cells.

### 3.3. rhIL-17B Can Reduce HECV Tubule Formation

**Figure 3:**
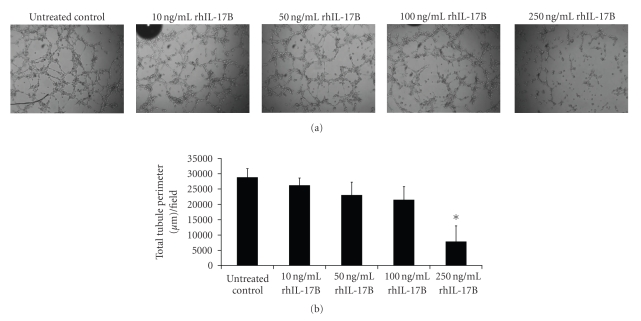
rhIL-17B inhibited HECV endothelial cell tubule formation. (a) Representative pictures of tubule formation following incubation with varying concentrations of rhIL-17B. (b) Histogram representing mean quantified total tubule length per field. A significant reduction was observed following treatment with 250 ng/mL rhIL-17B. *P < .05.

Untreated HECV endothelial cells formed tubule-like structures when seeded onto Matrigel demonstrating their angiogenic potential. Treatment of these cells with rhIL-17B adversely affected the capability of this cell line to form these tubule structures (Figure 3). This adverse effect was most notable at the highest concentration of 250 ng/mL where, following quantification of tubule perimeter, a significant decrease in tubules was observed in comparison to untreated control cells (*P* = .023). Whilst a significant reduction in tubule formation was only observed at the 250 ng/mL concentration, there does appear some marginal effects, following quantification, of rhIL-17B on HECV tubule formation at 100 ng/mL and possibly even at 50 ng/mL, where mean total perimeter length/field was generally reduced in comparison to the untreated control, though both of these treatments did not reach significance. Thus, this data suggests that IL-17B may negatively impact on the angiogenesis process through inhibiting formation of new vessels. This role for IL-17B seems to be in contrast with the proangiogenic effect of IL-17 observed by Numasaki et al., whose study demonstrated the capacity of IL-17 to enhance vascular endothelial cell cord formation over a range of concentrations in vitro and promote increased tumour vascularity in in vivo models [12]. In this study Numasaki et al. showed an enhancement of microvessel development at 50–500 ng/mL IL-17 concentrations. Whilst our study also tested a range of rhIL-17B concentrations, significant result were only observed at the higher dose (250 ng/mL) and little effect was observed at our lowest tested concentration of 10 ng/mL. However, IL-17B and its anti-angiogenic actions are not alone in the family. Other members of the IL-17 family have also been suggested to have anti-angiogenic influences at higher concentrations and an anti-angiogenic role for IL-17F was seen in a study by Starnes et al., which demonstrated the capacity of IL-17F to inhibit capillary tubule formation in an in vitro endothelial cell capillary tubule formation assay at concentrations of 100, 375, and 750 ng/mL [20]. Exerting biological functions at a higher concentration appears to be a common feature for the IL-17 family members. Another example is IL-17D, which has significant effects at a concentration range of 500–1000 ng/mL [21]. Thus, our data implies that IL-17B may function differently to IL-17 in angiogenesis or may itself not be acting as an independent angiogenic factor. Additionally, the high concentrations needed to bring about significant effects in vitro may limit the usefulness of IL-17B in vivo where physiological concentrations of this cytokine are likely to be substantially lower. Further investigation into both the mode of action of IL-17B together with additional studies examining the efficacy and toxicity of IL-17B over a range of concentration in vivo are required to fully realise the potential of this cytokine.

## 4. Conclusions

The data presented suggests that IL-17B may negatively impact on the angiogenic process through its ability to inhibit endothelial cell migration and tubule formation whilst having little effect on endothelial cell growth. Thus, it appears as though IL-17B could play an opposite role to that of IL-17 in the angiogenic process which, whilst also having little effect on endothelial cell proliferation, promoted endothelial cell migration and tubule formation [12], though direct comparisons between these two cytokines using similar methodologies are required to identify any contrasting roles. As far as the authors are aware, this data is the first implicating a role for IL-17B in the angiogenesis process. Additional work is required to fully investigate and establish the effects, either direct or indirect, of IL-17B in endothelial cells and to examine the efficacy of this cytokine to impact on angiogenesis using in vivo models. Thus, initial data suggests that IL-17B may hold potential as an anti-angiogenic therapeutic; however, the high concentrations needed to bring about any inhibition and how these would be tolerated in vivo must also be considered in future studies.
